# A magnesium efflux transporter required for seed development and eating quality in rice

**DOI:** 10.1073/pnas.2536813123

**Published:** 2026-04-22

**Authors:** Sheng Huang, Kiyosumi Hori, Naoki Yamaji, Yuma Yoshioka, Min Ning, Yu Nagaya, Takaaki Miyaji, Namiki Mitani-Ueno, Shin-ichiro Inoue, June-Sik Kim, Miho Kashino, Jian Feng Ma

**Affiliations:** ^a^College of Agronomy, Hunan Agricultural University, Changsha 410128, China; ^b^Institute of Plant Science and Resources, Okayama University, Kurashiki 710-0046, Japan; ^c^National Institute of Crop Science, National Agriculture Research Organization, Tsukuba, Ibaraki 305-8518, Japan; ^d^Graduate School of Medicine, Dentistry and Pharmaceutical Sciences, Okayama University, Kita, Okayama 700-8530, Japan; ^e^Department of Genomics and Proteomics, Advanced Science Research Center, Okayama University, Kita, Okayama 700-8530, Japan; ^f^Department of Regulatory Biology, Saitama University, Saitama 338-8570, Japan; ^g^RIKEN Center for Sustainable Resource Science, Tsurumi-ku, Yokohama 230-0045, Japan

**Keywords:** magnesium, rice, transporter

## Abstract

As a staple food for half the world’s population, rice is an important dietary source of magnesium (Mg), an essential mineral for human health. Enhanced Mg accumulation in rice grains has also been linked to eating quality. However, the mechanisms underlying Mg transport to the grains remains poorly understood. Here, we report that OsMGR2, a member belonging to Magnesium Release (MGR) family, is required for Mg accumulation in rice grains. *OsMGR2* encodes a plasma membrane-localized transporter that mediates Mg efflux. *OsMGR2* is constitutively and highly expressed in the stele tissues of roots, the phloem region of both enlarged and diffused vascular bundles in nodes, and the ovular vascular trace of caryopses. Knockout of this gene results in decreased root-to-shoot translocation and altered distribution of Mg to different organs; less Mg is allocated to the second newest leaf with high Mg requirement for active photosynthesis. The *osmgr2* mutants exhibit decreased Mg accumulation in the grain, which are smaller, lighter, and shriveled, but show increased accumulation in the husk. The eating quality of the mutant grains is significantly decreased compared with the wild-type rice. These results indicate that OsMGR2 plays multiple roles within the rice; facilitating the root-to-shoot Mg translocation, mediating phloem-to-xylem Mg transfer at nodes for preferential distribution to the most active leaf, and exporting Mg from maternal vascular tissues of the caryopsis to the grains, processes essential for grain development and eating quality in rice.

## Significance

Magnesium (Mg) is an essential element for both plants and humans. As a staple food, rice represents a major dietary source of Mg, yet the mechanism underlying Mg loading into grain remains unclear. In this study, we identified a Mg transporter, OsMGR2, which localizes to the plasma membrane and functions as an efflux transporter required for the seed development and eating quality in rice. Depending on its expression, OsMGR2 performs multiple roles; facilitating root-to-shoot Mg translocation, mediating phloem-to-xylem Mg transfer at nodes for preferential distribution to the most active leaf, and exporting Mg from maternal vascular tissues of the caryopsis to the grain.

Both plants and humans require large amounts of magnesium (Mg) as an essential element for growth and development. Mg is the second most prevalent cation in plants and participates in numerous biochemical and physiological processes, including photosynthesis, protein synthesis, and nucleotide metabolism (1). For instance, up to 35% of total Mg is allocated to the chloroplasts, where it forms the central atom of chlorophyll molecules (2); consequently, Mg deficiency leads to leaf chlorosis. Mg also serves as an essential cofactor for numerous enzymes, including photosynthetic enzymes and is a structural component of the ribosome (3).

In humans, Mg is the second most abundant cellular ion and the fourth most abundant ion in the human body (4). It is essential for energy metabolism, electrolyte balance, muscle contraction, and neurotransmission. As part of the Mg- Adenosine Triphosphate complex, it participates in over 600 enzymatic reactions. Mg deficiency results in symptoms such as fatigue, muscle cramps, and arrhythmias (5), and has also been associated with increased risks of coronary heart disease and metabolic disorders. Notably, hypomagnesemia is a common feature in type-2 diabetes (6, 7). Additionally, insufficient Mg supply from forage causes a physiological disorder known as grass tetany or grass staggers in ruminants (8, 9).

A healthy human body contains about 20 to 28 g Mg, of which 50 to 60% is stored in bone, 34 to 39% in muscles, soft tissues, and organs, and only 1 to 2% in blood and extracellular fluids (10). Therefore, a continuous supply of Mg with adequate amounts from daily diets is required for human health, as Mg in these skeletons is not easily mobilized when dietary Mg intake is reduced (9, 11). The recommended dietary allowance for Mg in adults is about 400 mg for males and 310 mg for females, increasing to 350 mg during pregnancy (12). To meet this requirement, dietary intake of Mg is required. Rice (*Oryza sativa* L.) is a staple food for half of the world’s population and the brown rice contains 1,121 to 2,029 mg Mg kg^−1^ with a mean of 1,558 mg Mg kg^−1^ depending on rice cultivars and growing environments (13). Although some Mg is lost during polishing, rice remains an important source of daily Mg intake. However, the mechanisms underlying Mg accumulation in rice grains remain poorly understood.

Similar to other mineral elements essential for plant growth, the transfer of Mg from soil to the rice grains involves multiple steps, including at least uptake by roots from soil, translocation from roots to above-ground parts, distribution and redistribution between different organs and tissues, and final delivery to grains (13). Recently, it was reported that the last step is mainly mediated by transporters localized at nodes in rice plants, particularly node I (the uppermost node), which connects with the flag leaf and panicle (14). Several node-expressed transporters involved in accumulation of mineral elements in rice grains have been identified. For example, SULTR-like phosphorus distribution transporter, a phosphorus (P) transporter expressed in the node I, is responsible for delivery of P to the rice grain (15). Three silicon (Si) transporters; Low silicon 2/3/6 (Lsi2, Lsi3, and Lsi6)-localized to different cell layers of node I are required for Si distribution to the rice grains (16).

In the present study, we found that OsMGR2, belonging to Magnesium Release (MGR) transporter family, shows relatively high expression in the node I. Through detailed functional characterization of this gene, we revealed that OsMGR2 plays multiple roles in Mg transport within rice, including root-to-shoot translocation, preferential distribution to the most active leaf, and export of Mg from maternal vascular tissues of the caryopsis-processes required for both grain development and eating quality.

## Results

### Expression Pattern of *OsMGR2* at Different Growth Stages.

Node I in rice serves as a hub for distribution of mineral elements to the grains (14, 15). To identify genes involved in Mg distribution, we searched the gene expression profiles of node I from our previous studies (16–18), and found that *OsMGR2* (*Os03g0125800*) belonging to the MGR transporter family, showed relatively high expression in the node I. We therefore first investigated the expression pattern of *OsMGR2* across different organs and growth stages.

**Fig. 1.**
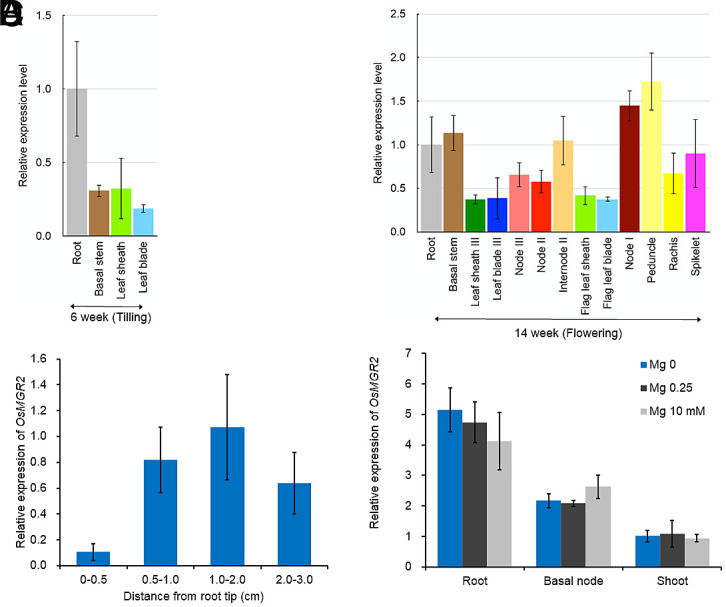
Expression pattern of *OsMGR2*. (*A* and *B*) Growth stage- and organ-dependent expression of *OsMGR2*. Samples of various organs were collected at vegetative (*A*) and reproductive (*B*) stages from rice grown in the field. (*C*) Spatial expression pattern of *OsMGR2* in roots. Different root segments, as indicated, were collected from 5-d-old seedlings. (*D*) Response of *OsMGR2* expression to different Mg concentrations. 25-d-old seedlings (cv. Nipponbare) were exposed to a solution containing 0, 0.25, or 10 mM Mg for 7 d. Roots, shoot basal regions, and shoots were then sampled for RNA extraction. The expression level of *OsMGR2* was determined by RT-qPCR with *Histone H3* used as an internal control. Expression is shown relative to root (*A* and *B*), root segment of 1.0 to 2.0 cm (*C*), and shoot under 0 mM Mg condition (*D*). Data represent means ± SD of three (*A* and *B*) or four (*C* and *D*) biological replicates (n = 3 or 4).

At the vegetative growth stage (tillering stage), *OsMGR2* was ubiquitously expressed in all organs tested, with a greater expression in the roots (Fig. 1*A*). At the reproductive growth stage (flowering stage), greater expression of *OsMGR2* was found in the nodes, especially node I (Fig. 1*B*). Spatial analysis of the roots showed that *OsMGR2* was more highly expressed in the root mature region (1 to 2 cm) compared with the root tip (Fig. 1*C*). Furthermore, we examined the response of *OsMGR2* to Mg deficiency (0 mM) and excess (10 mM). The expression of *OsMGR2* was not altered by Mg deficiency or excess in the roots, shoot basal region, or leaves (Fig. 1*D*), indicating that *OsMGR2* is constitutively expressed in all these organs.

### Subcellular and Cellular Localization of OsMGR2.

To observe the subcellular localization of OsMGR2, we transiently expressed *Green Fluorescent Protein* (*GFP*) fused to the N-terminus of *OsMGR2* in rice leaf sheath protoplasts. The fluorescence signal of cell expressing *GFP* alone was observed in the cytosol and nucleus (*SI Appendix*, Fig. S1), overlapping with the DsRed signal (cytosol and nucleus marker). By contrast, the signal of GFP-OsMGR2 was observed at the periphery of the cell and did not overlap with the DsRed signal (*SI Appendix*, Fig. S1), indicating that OsMGR2 is a plasma membrane-localized protein.

**Fig. 2.**
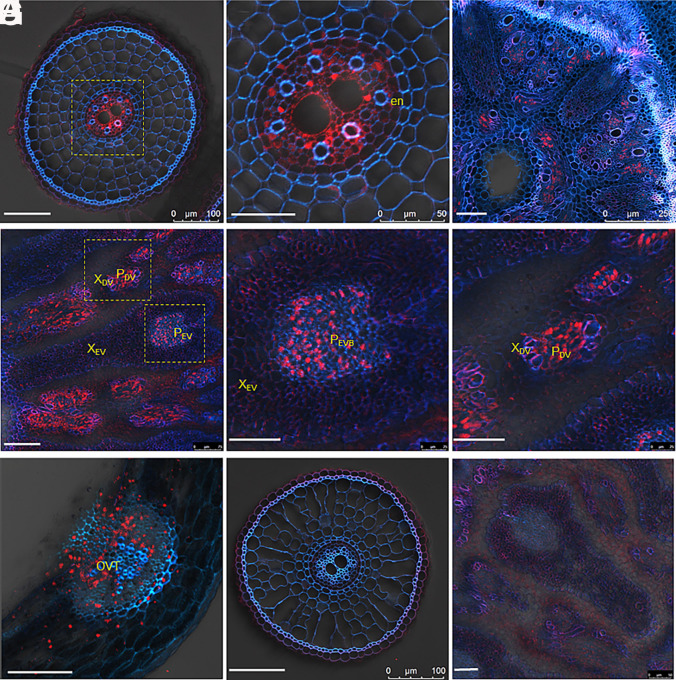
Tissue specificity of OsMGR2 in rice. (*A*–*C*) Tissue localization of OsMGR2 at the vegetative stage in root (*A* and *B*) and basal node (*C*) of transgenic lines carrying the *OsMGR2* promoter-*GFP*. 21-d-old seedlings of the transgenic lines were used. (*B*) Enlarged image of yellow dotted box in (*A*). (*D–F*) Tissue localization of OsMGR2 in node I at the reproductive stage. Node I was sampled from soil-grown rice at the grain filling stage. (*E* and *F*) Enlarged image of yellow dotted box in (*D*). (*G*) Tissue localization of OsMGR2 in caryopses. (*H* and *I*) Root (*H*) and node I (*I*) of nontransgenic line serving as negative controls. Immunostaining with GFP antibody was performed. Red color indicates the GFP antibody-specific signal; blue color indicates cell wall autofluorescence. Images are representative of more than 10 cross sections. [Scale bar, 100 µm in (*A*, *C*, *D*, *G*, and *H*) and 50 µm in (*B*, *E*, *F*, and *I*).] Abbreviations: en, endodermis; X_EV_, xylem of enlarged vascular bundle; P_EV_, phloem of enlarged vascular bundle; X_DV_, xylem of diffuse vascular bundle; P_DV_, phloem of enlarged vascular bundle; OVT, ovular vascular trace.

To investigate the tissue- and cell-specific expression of *OsMGR2*, we generated transgenic rice lines expressing *GFP* under the control of the *OsMGR2* promoter. At the vegetative stage, immunostaining with GFP antibody revealed that *OsMGR2* was mainly expressed in the stele region of rice roots as well as the phloem region of the basal node (Fig. 2 *A–C*). At the reproductive stage, GFP signal was mainly detected in the phloem region of both enlarged vascular bundles (EVB) and diffuse vascular bundles (DVB) of node I (Fig. 2 *D–F*). To confirm this result, we extracted the expression pattern of *OsMGR2* from laser microdissection (LMD) combined RNA-seq in node I and compared it with *Oryza sativa* **Oligo-Peptide Transporter* (*OsOPT7*)* as a control. *OsOPT7* encodes a ferrous iron transporter (17) and is mainly expressed in the xylem parenchyma cells of the EVB (17). By contrast, *OsMGR2* was highly expressed in the phloem regions of node I (*SI Appendix*, Table S1), consistent with the immunostaining results (Fig. 2 *D–F*).

In the caryopses, *OsMGR2* was expressed at the ovular vascular trace (OVT) (Fig. 2*G*). To confirm this expression pattern, we also extracted the tissue-specific expression profile based on LMD combined RNA-seq analysis in immature grain (*SI Appendix*, Table S2) using *Oryza sativa* *Vacuolar Iron Transporter2* (*OsVIT2*) as a control. *OsVIT2* encodes a ferrous iron transporter (18) and is mainly expressed in the aleurone layer and radicle of the caryopsis (18). Unlike this expression pattern, *OsMGR2* was mainly expressed in the OVT (*SI Appendix*, Table S2), confirming the immunostaining results (Fig. 2*G*). No signal was detected in the nontransgenic plants (Fig. 2 *H* and *I*), indicating the high specificity of this antibody.

### Transport Activity of OsMGR2.

**Fig. 3.**
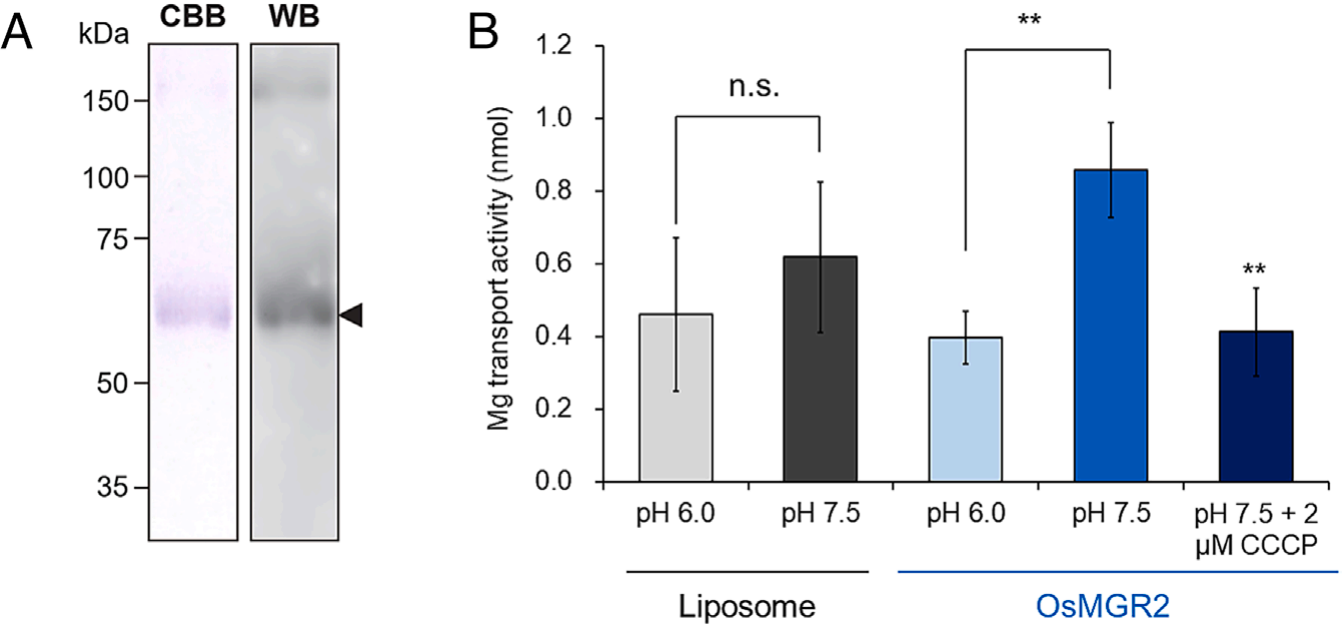
Transport activity of OsMGR2 protein. (*A*) Purification of OsMGR2 proteins. The purified fraction was analyzed by SDS–PAGE and visualized by Coomassie Brilliant Blue staining (*Left*) and by western blot with anti-6× His antibody (*Right*). (*B*) OsMGR2-mediated transport activity. Proteoliposome containing OsMGR2 were incubated in a buffer solution containing 1 mM Mg at either pH 6.0 or pH 7.5, or at pH 7.5 with 2 μM CCCP, and assayed after 2 min. Mg concentration in liposomes was determined by ICP-MS. Data represent means ± SD from independent experiments (n = 5 to 6). **P < 0.01 (two tailed paired Student’s *t* test). Results at pH 7.5 were compared with those at pH 7.5 + CCCP.

To investigate the transport activity of OsMGR2 for Mg, we employed a proteoliposome-based transport assay system. *OsMGR2* was expressed in insect cells using recombinant baculovirus. The purified protein fraction exhibited a major protein band at the expected molecular weight (56.5 kDa) of OsMGR2 (Fig. 3*A*), which was confirmed by western blot with anti-6× His antibody (Fig. 3*A*). Transport assays showed that proteoliposomes containing recombinant OsMGR2 exhibited significantly higher transport activity for Mg when a pH gradient was present (outside pH 7.5 vs. inside pH 6.0, mimicking cytosol vs. apoplast) (Fig. 3*B*). However, no transport activity was detected in control liposomes (without OsMGR2 protein), even with a pH gradient (Fig. 3*B*). Furthermore, the transport activity was abolished in the presence of 2 μM carbonyl cyanide m-chlorophenylhydrazone (CCCP), a protonophore. These results indicate that OsMGR2 functions as an efflux transporter of Mg^2+^ coupled with H^+^.

### Phenotypic Analysis of *osmgr2* Mutants at Vegetative Growth Stage.

To examine the physiological role of *OsMGR2*, we generated knockout lines of *OsMGR2* using CRISPR/Cas9-mediated targeted mutagenesis. We obtained two independent mutant lines, *osmgr2-1* and *osmgr2-2*, each with a single base insertion (thymine or cytosine) in the first exon, resulting in frameshift and loss of function of the target gene (*SI Appendix*, Fig. S2*A*). The expression level of the mutated gene was also lower in the mutants (*SI Appendix*, Fig. S2*B*).

**Fig. 4.**
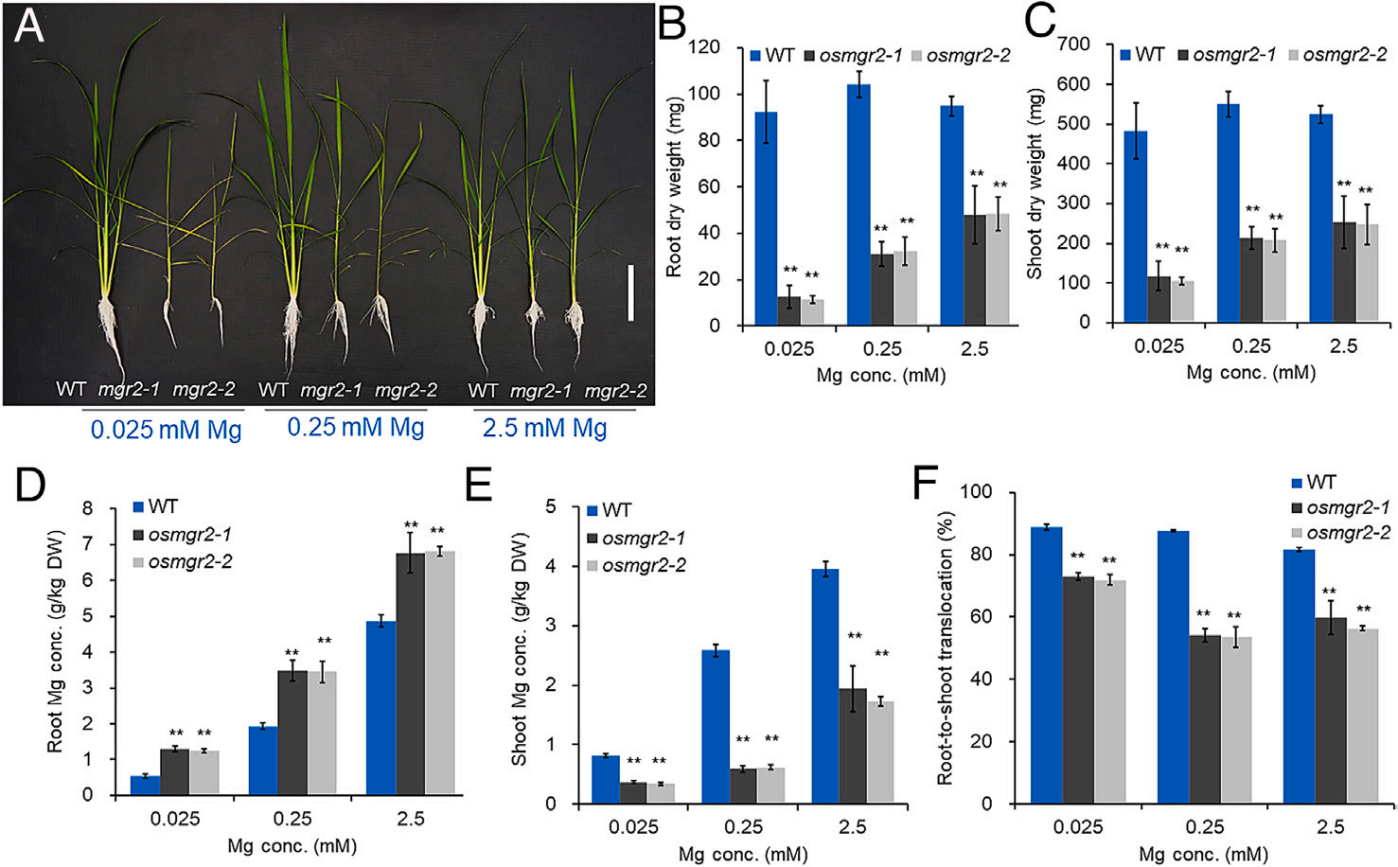
Effect of knockout of *OsMGR2* on growth, and Mg accumulation at the vegetative stage. (*A*) Growth of the WT and mutant plants under different Mg concentrations. (Scale bar, 10 cm.) (*B* and *C*) Root (*B*) and shoot (*C*) dry weight. (*D* and *E*) Mg concentration in roots (*D*) and shoots (*E*). (*F*) Root-to-shoot translocation of Mg. 16-d-old plants precultured at 2.5 mM Mg for 16 d were exposed to nutrient solutions containing 0.025, 0.25, and 2.5 mM for 17 d. Data represent means ± SD of four biological replicates (n = 4). ** indicates significant difference compared with WT (*P* < 0.01, one-way ANOVA followed by Tukey’s test).

When these lines and wild-type (WT) rice were grown in a solution containing different Mg concentrations (from 0.025 to 2.5 mM) for 17 d, severe growth retardation was observed in mutants at low Mg concentration (0.025 mM) (Fig. 4*A*), and the leaves of the mutants-but not the WT-showed severe chlorosis. With increasing Mg supply to 2.5 mM, the mutant growth partially recovered (Fig. 4*A*), but remained poorer compared with WT (Fig. 4*A*). Root dry weight of mutants was 49 to 88% of WT, and shoot dry weight was 52 to 78% of WT across different Mg supplies (Fig. 4 *B* and *C*).

In a separate experiment, by using the Soil Plant Analysis Development (SPAD) chlorophyll meter, we compared SPAD values between WT and two knockout lines under different Mg concentrations. Under low Mg condition (0.01 mM), the newest fully expanded leaf of knockout lines, but not of WT, showed more severe chlorosis symptoms (*SI Appendix*, Fig. S3*A*). At all Mg concentrations tested, SPAD values were consistently lower in the mutants than in the WT (*SI Appendix*, Fig. S3*B*). Particularly at low Mg concentration (0.01 mM), SPAD values were only around 20 in the mutants, compared with >30 in the WT. SPAD values in the mutants increased with increasing Mg supply, indicating that lower SPAD values in the mutants result from Mg deficiency.

Analysis of mineral elements showed that Mg concentration was increased in the roots but decreased in the shoots of the mutants compared with the WT (Fig. 4 *D* and *E*), resulting in decreased root-to-shoot translocation of Mg (Fig. 4*F*). By contrast, no consistent difference in other mineral elements was observed between the WT and *osmgr2* mutants, although accumulation of some elements was also altered. For example, the concentration of P, Ca, Fe, Cu, and Zn was increased in roots of knockout lines compared with the WT at either Mg treatment concentration (*SI Appendix*, Fig. S4), while the concentration of K, Ca, Mn, Fe was increased in shoots of knockout lines at some Mg treatment concentrations (*SI Appendix*, Fig. S5). These altered accumulations of other elements are possibly an indirect consequence of decreased Mg accumulation and growth inhibition in mutants (Fig. 4).

### Short-Term Labeling Experiment with ^25^Mg at the Vegetative Stage.

To confirm above results (Fig. 4), we performed a short-term (48 h) labeling experiment with stable isotope ^25^Mg. The labeling experiment was conducted by exposing the plants to a nutrient solution containing 25 μM ^25^Mg, in the presence of 1 μM rubidium (Rb) and strontium (Sr) for 2 d. Rb and Sr were used as symplastic and apoplastic tracers, respectively. Similar to the results in Fig. 4, the concentration of ∆^25^Mg in the roots was greater in the mutants than in the WT, whereas that in the shoots was lower in the mutants compared with the WT (*SI Appendix*, Fig. S6*A*), resulting in lower root-to-shoot translocation of ∆^25^Mg in the mutants (*SI Appendix*, Fig. S6*B*). By contrast, although the concentration of Rb and Sr was also altered in the mutants probably due to inhibited growth (*SI Appendix*, Fig. S6 *C* and *D*), unlike ∆^25^Mg, the concentration of Rb and Sr was increased in the shoot of the mutants compared with the WT.

Organ-dependent analysis showed that ∆^25^Mg concentration was decreased in all above-ground organs due to the decreased root-to-shoot translocation of Mg (*SI Appendix*, Fig. S7*A*). However, the distribution ratio analysis showed that ∆^25^Mg distributed to the shoot basal region including basal nodes was increased, whereas that distributed to the developed leaves (leaf 2 to 6) was decreased in the mutants compared with the WT (*SI Appendix*, Fig. S7*B*). More than 30% of total ∆^25^Mg was distributed to the leaf 7 of the WT (the second newest leaf, with active photosynthesis), whereas less than 20% was distributed to the same position in the mutants (*SI Appendix*, Fig. S6*B*). Surprisingly, distribution to the leaf 8 (the newest leaf) was increased in the mutants compared with the WT.

### Phenotypic Analysis of *osmgr2* Mutants at Reproductive Growth Stage.

**Fig. 5.**
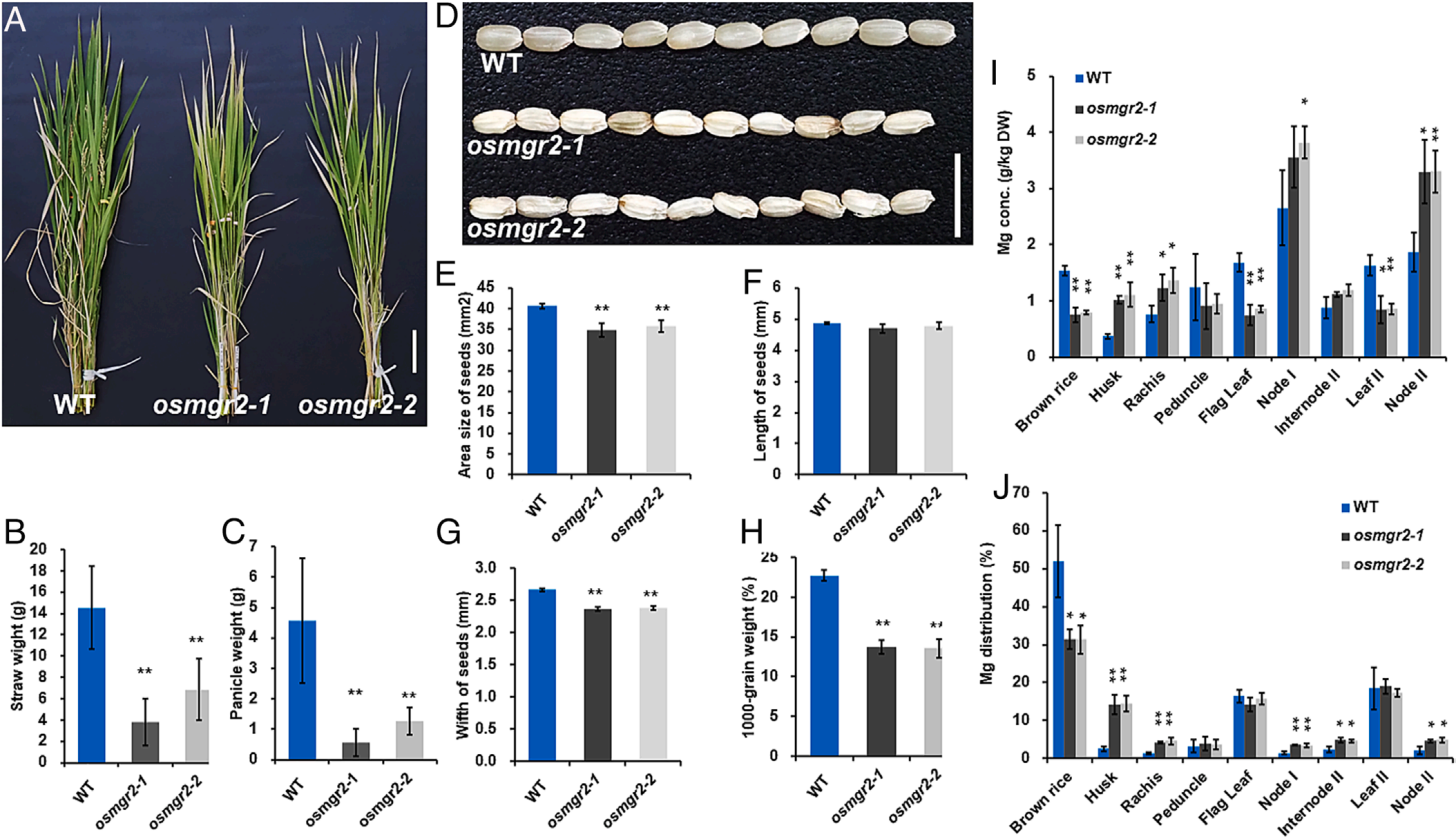
Effect of *OsMGR2* knockout on rice productivity, seed development, and Mg accumulation at the reproductive stage. (*A*) Phenotype of WT rice and mutants grown in soil at harvest. (Scale bar, 10 cm.) (*B* and *C*) Dry weight of straw (*B*) and panicles (*C*). (*D*) Phenotype of brown rice of WT and mutants. (Scale bar, 1 cm.) (*E* to *G*) Grain size (*E*), grain length (*F*), and grain width (*G*). (*H*) Grain weight. (*I* and *J*) Concentration (*I*), and distribution (*J*) of Mg in different organs. Both the WT and knockout lines were grown in pot soil under flooded conditions until maturity. Data represent means ± SD of three biological replicates (n = 3; 200 seeds were measured for each replicate in *E*–*G*). * or ** indicates significant difference compared with WT (*P* < 0.05 or *P* < 0.01, one-way ANOVA followed by Tukey’s test).

Since *OsMGR2* is expressed in caryopses, we grew the mutants and WT in pot soil until maturity to examine the role of *OsMGR2* in grain development. Knockout of *OsMGR2* resulted in poor growth (Fig. 5*A*). The weight of straw and panicles of the mutants were only 34% and 22% of the WT (Fig. 5 *B* and *C*), respectively. The grains of the mutants showed opaque color and decreased transparency compared with the WT (Fig. 5*D*). Moreover, the grain of the *osmgr2* mutants were shriveled (Fig. 5*D*). The area size of the grains of the mutants was 86 to 88% of the WT (Fig. 5*E*). The length of the grain did not differ between WT and mutants (Fig. 5*F*), whereas the width of the grain was shorter in the mutants than in the WT (Fig. 5*G*). Furthermore, the grain weight of the mutants was much lower than that of WT (Fig. 5*H*).

Analysis of Mg in different organs showed that Mg concentration in the brown rice was greatly decreased in the mutants, but increased in the husk, node I, and node II compared with the WT (Fig. 5*I*). The distribution ratio of Mg to different organs was calculated and compared between mutants and WT. In *osmgr2* mutants, less Mg was delivered/reached to the grain, but more Mg was distributed/retained to the node I and internode II compared with WT (Fig. 5*J*). However, no consistent difference in the distribution of other elements including P, K, Ca, Mn, Fe, Cu, and Zn was found between mutants and WT, although the distribution of some elements also differed in specific organs (*SI Appendix*, Fig. S8).

### Short-Term Stem-Feeding Experiment with Stable Isotope ^25^Mg.

To investigate the direct role of node-expressed *OsMGR2* in Mg distribution to the grain, we performed a short-term (24 h) stem-feed experiment with the stable isotope ^25^Mg. ^25^Mg was fed from the cut end at the internode III (below the node II) and distribution to different organs was compared between mutants and WT. Results showed that the distribution of ∆^25^Mg to the spikelet was decreased in the mutants compared with the WT (*SI Appendix*, Fig. S9*A*), whereas more Mg was distributed to the node I. As a control, no consistent differences in the distribution of Rb and Sr were found between mutants and WT (*SI Appendix*, Fig. S9 *B* and *C*). This distribution pattern is consistent with that observed in the long-term pot experiment (Fig. 5*J*).

### Comparison of Cooked Eating Quality between WT and *Osmgr2* Mutants.

**Fig. 6.**
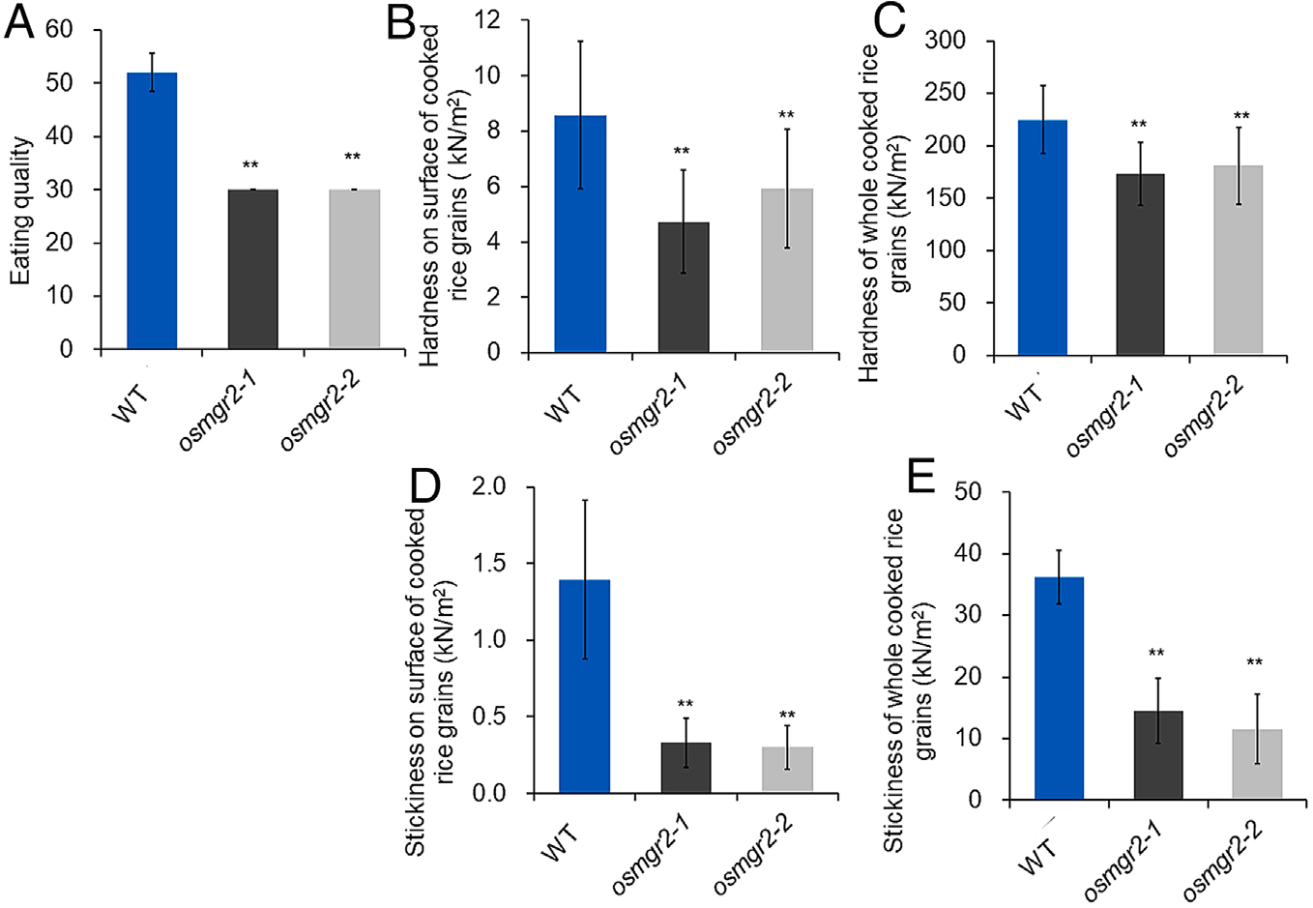
Effect of *OsMGR2* knockout on eating quality of rice. (*A*) Eating quality score. (*B* and *C*) Hardness of cooked rice grain surface (*B*) and whole cooked rice grains (*C*). (*D* and *E*) Stickiness of cooked rice grain surface (*D*) and whole cooked rice grains (*E*). Seeds harvested from both the WT rice and two knockout lines (*osmgr2-1*, *osmgr2-2*) grown in pot soil were dehulled and subjected to eating-quality tests using a taste analyzer and Tensipresser. Data represent means ± SD of three biological replicates (n = 3). ** indicates significant difference compared with WT (*P* < 0.01, one-way ANOVA followed by Tukey’s test).

In addition to nutritional quality such as trace element density, cooked eating quality is also a very important trait for commercial value of rice grains. Since knockout of *OsMGR2* decreased grain transparency (Fig. 5*B*), we investigated whether it would affect the cooked eating quality. To evaluate eating quality, we used two instrumental methods; Cooked Rice Taste Analyser and Tensipresser, which are significantly correlated with eating quality scores by the sensory test. The eating quality score was significantly lower in the mutants than in the WT (Fig. 6*A*). Hardness and stickiness of the cooked grains were also significantly reduced in the mutants compared with the WT. (Fig. 6 *B–E*). These results indicate that knockout of *OsMGR2* decreased the eating quality.

### RNA-seq Analysis of Immature Grains.

To understand the mechanism underlying low Mg accumulation-inhibited grain development and Mg homeostasis, we performed an RNA-seq analysis using immature grains of both the WT and mutant. The RNA-seq data have been deposited in DDB BioProject (PRJDB40261) (19). Compared with the WT, a total of 621 differentially expressed genes (DEGs) were identified in the *osmgr2* mutant [≧twofold; false discovery rate < 0.05]. Among them, 348 genes were significantly up-regulated and 273 genes were significantly down-regulated (Dataset S1), while no Mg transport-related genes were included. These DEGs were further subjected to Gene Ontology (GO) term enrichment analysis and Kyoto Encyclopedia of Genes and Genomes pathway enrichment analysis to systematically explore the biological processes and metabolic pathways affected by knockout of *OsMGR2* gene during the grain development. Enrichment analysis of the up-regulated genes in the mutant showed that these genes were enriched in multiple biological processes, such as ribonucleoprotein complex biogenesis, ribosome biogenesis, gene expression, translation as well as in the pathways of ribosome and ribosome biogenesis in eukaryotes (*SI Appendix*, Fig. S10 *A* and *C*). This could be attributed to Mg functioning as a structural component of ribosomes, where it is essential for activating amino acid polymerization into polypeptide chains and for protein biosynthesis (20). On the other hand, genes down-regulated in the *osmgr2* mutant were enriched in multiple biological processes, such as negative regulation of catalytic activity, negative regulation of molecular function, negative regulation of peptidase activity as well as in the pathways of protein processing in endoplasmic reticulum and starch and sucrose metabolism (*SI Appendix*, Fig. S10 *B* and *D*). Since Mg^2+^ is an essential cofactor for some enzymes of starch synthesis, insufficient supply of Mg^2+^ leads to decreased enzyme activity, thereby inhibiting starch synthesis and grain development. Therefore, these transcriptomic changes are most likely second consequences of Mg deficiency due to loss of *OsMGR2* function.

## Discussion

Both grain development and eating quality are important traits for rice production. Grain development directly determines rice yield, while eating quality relates to market value and consumer preference. In the present study, we found that OsMGR2 plays an important role in grain development and eating quality by transporting Mg to the shoot, mediating preferential Mg distribution to active leaves, and facilitating Mg delivery to the grains in rice (Figs. 4–6).

OsMGR2 belongs to a newly discovered family of Mg transporters known as ancient conserved domain proteins, which have been found to transport Mg in prokaryotes, fungi, animals, and plants (21–26). In plants, this family is specifically designated as MGR (Magnesium Release Transporter). The *Arabidopsis* genome contains nine MGR family members (AtMGR1-AtMGR9), divided into three clades. AtMGR1, AtMGR2, and AtMGR3 (clade I) localize to the tonoplast and are involved in vacuolar sequestration of Mg (23, 24), while AtMGR4, AtMGR5, AtMGR6, and AtMGR7 (clade II) localize to the plasma membrane and mediate root-to-shoot translocation of Mg (22). The remaining two members, AtMGR8 and AtMGR9 (clade III), localize to the inner envelope of the chloroplast and facilitate chloroplast Mg uptake (27). By contrast, the rice genome contains only four MGR members (OsMGR1–OsMGR4). Among these, only OsMGR3 belonging to clade II has been functionally characterized in terms of Mg transport (28). *OsMGR3* is highly expressed in the aleurone layer of the endosperm. Although knockout of this gene did not affect the growth, the seed germination was impaired in the mutants, indicating that OsMGR3 is involved in transporting Mg from the aleurone layer to the embryo during germination (28). In the present study, we found that OsMGR2 is involved in root-to-shoot translocation, preferential distribution to the most active leaf, and export of Mg from maternal vascular tissues of the caryopsis.

*OsMGR2* encodes a plasma membrane-localized efflux transporter for Mg (Fig. 3 and *SI Appendix*, Fig. S1). The closest homolog of OsMGR2 in *Arabidopsis* is AtMGR4 in clade II (24). At the vegetative growth stage, OsMGR2 is highly expressed in the root stele region (Fig. 2), and knockout of this gene resulted in increased Mg accumulation in the roots, but decreased Mg accumulation in the shoots at all Mg concentrations tested (Fig. 4). These findings indicate that similar to AtMGR4, OsMGR2 expressed in the root stele region is required for the root-to-shoot translocation of Mg.

Unlike other elements, Mg taken up by the roots show a distinct distribution pattern (29), characterized by preferential distribution to the second newest leaf, which is expanding and has high Mg requirements for active photosynthesis. We found that OsMGR2 expressed in the nodes plays a crucial role in this process. Normally, large amounts of Mg are loaded into the phloem of vascular tissues, which directs Mg to sink organs. At the vegetative stage, the phloem flow is directed to the newest developing leaf, but not to the second newest leaf (newly developed), while at the reproductive stage, phloem flow is directed to the grains. However, Mg must also be transported to the second newest leaf for the active photosynthesis at vegetative stage, and to tissues including peduncle, rachis, and green grains for panicle development at the reproductive stage. Therefore, to preferentially distribute Mg to these tissues, Mg must be reloaded from phloem to xylem in the nodes, which connects to the second newest leaf and green panicles. OsMGR2 expressed in the phloem region of DVB would function as an efflux transporter for phloem-xylem transfer of Mg. This is supported by the finding that knockout of *OsMGR2* decreased Mg to the second newest leaf but increased distribution to the newest leaf (*SI Appendix*, Fig. S7*B*).

OsMGR2 is also expressed in the caryopsis (Fig. 3*G* and *SI Appendix*, Table S2). Mineral elements passing through node I are finally unloaded into the grain from the OVT, the region of entry into the grain (30, 31). OsMGR2 expression in the OVT suggests its role in delivering Mg from the maternal tissues to the filial tissues (the aleurone and endosperm) (Fig. 3*G*). This is supported by the finding that knockout of *OsMGR2* resulted in high Mg accumulation in the husk, but lower Mg accumulation in the brown rice (Fig. 5). The grains of *osmgr2* mutants showed a shrunken phenotype with lighter weight and smaller size (Fig. 5), indicating that Mg in the grains plays a crucial role in their development. Grain development requires active synthesis of starch and proteins, which is an energy-intensive process (32, 33). Mg binds to ATP, forming the Mg–ATP complex—the biologically active form used by enzymes, including those involved in starch and protein synthesis. Therefore, lack of Mg slows down these synthesis pathways, resulting in development of grains with lower starch content (affecting weight) and lower protein content (affecting quality). Indeed, our RNA-seq analysis showed that expression level of genes related to starch synthesis is down-regulated in the mutant (*SI Appendix*, Fig. S10). Therefore, the reduced eating quality in *osmgr2* mutants likely results from a combination of Mg limitation and indirect developmental effects.

Eating quality of rice is a complex trait influenced by many factors (34–36). One such factor is Mg accumulation in the grains. It has been reported that high Mg content is associated with good eating quality (37). In the present study, we found that knockout of *OsMGR2* negatively affects the eating quality parameters including stickiness, firmness, and softness (Fig. 6). This may be attributed to the roles of Mg in enzymes involved in starch and protein content that indirectly affect eating quality, although further studies are required.

In conclusion, we found that OsMGR2 plays multiple roles in Mg transport in rice. OsMGR2 expressed in the root stele region is responsible for the root-to-shoot translocation of Mg; OsMGR2 expressed in the nodes is required for preferentially delivering Mg to the second newest organs; and OsMGR2 expressed at the OVT is responsible for exporting Mg from maternal vascular tissues of the caryopsis to the grain, processes crucial for grain development and eating quality in rice (*SI Appendix*, Fig. S11). Controlling Mg accumulation is not only important for improving eating quality in rice grains but also to enhance nutritional quality for humans.

## Materials and Methods

WT rice (WT, cv. Nipponbare) and two independent knockout lines of *OsMGR2* (*osmgr2-1* and *osmgr2-2*, T_2_/T_3_ generations) were used for phenotypic analysis in both hydroponic and soil culture. Gene expression was determined by real-time RT-PCR and the tissue localization was investigated by immunostaining. Transport activity of OsMGR2 was examined using proteoliposomes. Eating quality score was measured with a Cooked Rice Taste Analyser STA1A (Satake Co. Ltd.) and physical properties of cooked grains were measured with a Tensipresser MyBoy texture analyser (Takemoto Electric Co.). Concentrations of mineral elements in the digested solution were determined by ICP-MS. For more details, see **SI Appendix*, Materials and Methods*.

## Supplementary Material

Appendix 01 (PDF)

Dataset S01 (XLSX)

The SI Appendix has been updated to correct the legends for Figures S3-S5.

## Data Availability

RNA-seq data have been deposited in DDB BioProject (PRJDB40261) (19). All other data are included in the manuscript and/or supporting information.
