# Supplementary material for: A magnesium efflux transporter required for seed development and eating quality in rice
Source: Proc Natl Acad Sci U S A. 2026 Apr 22;123(17):e2536813123. doi: 10.1073/pnas.2536813123 (PMC13123918; doi:10.1073/pnas.2536813123)
Supplement: Supplementary file 1 — Appendix 01 (PDF) [file pnas.2536813123.sapp.pdf]

Supporting information for

A magnesium efflux transporter required for seed development and eating quality in rice

Sheng Huang[1,2], Kiyosumi Hori[3], Naoki Yamaji[2], Yuma Yoshioka[4], Min Ning[2], Yu Nagaya[4], Takaaki Miyaji[4,5], Namiki Mitani-Ueno[2], Shin-ichiro Inoue[6], June-Sik Kim[2,7], Miho Kashino[2], and Jian Feng Ma[2]*

[1] College of Agronomy, Hunan Agricultural University, Changsha 410128, China
[2] Institute of Plant Science and Resources, Okayama University, Chuo 2-20-1, Kurashiki 710-0046, Japan
[3] National Institute of Crop Science, National Agriculture Research Organization, 2-1-2 Kannondai, Tsukuba, Ibaraki 305-8518, Japan
[4] Graduate School of Medicine, Dentistry and Pharmaceutical Sciences, Okayama University, Tsushima Naka 1-1-1, Kita, Okayama, 700-8530, Japan
[5] Department of Genomics & Proteomics, Advanced Science Research Center, Okayama University, Tsushima Naka 1-1-1, Kita, Okayama, 700-8530, Japan
[6] Department of Regulatory Biology, Saitama University, Saitama 338-8570, Japan
[7] RIKEN Center for Sustainable Resource Science, 1-7-22 Suehiro-cho, Tsurumi-ku, Yokohama, 230-0045 Japan

* To whom correspondence should be addressed: E-mail: maj@rib.okayama-u.ac.jp

This PDF file includes:

Supplementary text
Figures S1 to S11
Tables S1 to S3
SI references

Materials and Methods

Plant materials and growth conditions

Wild-type rice (WT, cv. Nipponbare) and two independent knockout lines of *OsMGR2* (*osmgr2-1* and *osmgr2-2*, T_2/T_3 generations) generated as described below were used in this study. De-husked seeds were sown in half strength Kimura B at 30 ℃, in the dark for 2 days, then transferred onto a net floating on the nutrient solution. At day 7, seedlings were transferred to 3.5-L of nutrient solution in plastic pots. For preculture, 2.5 mM Mg was added to the solution to improve mutant growth (pH 5.6). The nutrient solution was renewed every 2 days (1). Plants were grown in a controlled glasshouse at 25-30ºC under natural light and used for the following analysis. All experiments were performed with three to four biological replicates.

Generation of *OsMGR2* knockout lines by CRISPR/Cas9

Knockout lines of *OsMGR2* were generated using the CRISPR/Cas9 technique. Twenty bases upstream of the protospacer adjacent motif (PAM) were selected as candidate target sequences (*SI Appendix*, Fig. S2). Primers for the target sequences in the first exon are listed in *SI Appendix*, Table S3. Double-stranded target fragments were generated by annealing complementary single-stranded oligonucleotides after denaturation at 95°C for 5 min. The annealed products were subsequently inserted into the *Bbs*I-digested pU6gRNA vector. The OsU3-gYSA cassette in pZDgRNA-Cas9ver.2_HPT was then substituted with the synthesized gRNA expression module via *Asc*I and *Pac*I restriction sites (2). The resulting construct was transformed into rice (cv. Nipponbare) calluses according

to Hiei et al. (3).

To genotype the resulting mutants, genomic DNA was extracted from leaves of transgenic lines, followed by polymerase chain reaction (PCR) amplification using primer pairs flanking the designed target sites (*SI Appendix*, Table S3). PCR products (approximately 500 bp) were directly sequenced using internal specific primers (*SI Appendix*, Table S3). Two homologous knockout lines (*osmgr2-1* and *osmgr2-2*) without T-DNA were selected for subsequent phenotypic analysis. The expression of *OsMGR2* in the roots in these mutant lines and WT was determined by the method described below.

Expression pattern analysis of *OsMGR2*

To investigate the expression pattern of *OsMGR2* in different organs at various growth stages, we used the same cDNA samples which was prepared in a previous study (4). To examine the response of *OsMGR2* expression to different Mg concentrations in different organs (root, shoot basal region, and shoot), 25-d–old seedlings (cv. Nipponbare) were exposed to nutrient solution containing 0, 0.25, or 10 mM Mg. After 7 days, the roots, shoot basal regions, and shoots were sampled for RNA extraction. For spatial expression analysis, different root segments (0–0.5, 0.5–1.0, 1.0–2.0, 2.0–3.0 cm from the root tip) were excised from the roots of 5-d-old seedlings.

Collected samples were immediately frozen in liquid nitrogen, and total RNA was extracted using a RNeasy Plant Mini Kit (Qiagen). cDNA was synthesized by ReverTra Ace qPCR RT Kit (TOYOBO) or SuperScript II (Invitrogen) according to the manufacturer’s instructions. Gene expression was determined using

SsoAdvanced Universal SYBR Green Supermix (Bio-Rad) or KOD SYBR qPCR Mix (TOYOBO) on a CFX384 or CFX96 real-time PCR machine (Bio-Rad). *Histone H3* was used as an internal control. Relative gene expression was calculated using the $2^{-\Delta\Delta Ct}$ method. Three or four independent biological replicates were performed for each treatment. Primer sequences are listed in *SI Appendix*, Table S3.

Subcellular localization of OsMGR2

The subcellular localization of OsMGR2 was investigated by transiently expressing a *GFP-OsMGR2* fusion or *GFP* alone into rice protoplasts, with DsRed serving as a cytosolic and nuclear marker. The open reading frame (ORF) of *OsMGR2* was amplified by PCR from rice (cv. Nipponbare) leaf cDNA using the primers listed in *SI Appendix*, Table S3. The ORF was inserted into a cauliflower mosaic virus 35S GFP vector at the N terminus according to Sasaki et al. (5). Rice protoplasts were prepared from leaf sheath of 2-week-old seedlings grown hydroponically. The polyethylene glycol method was used for transformation (6). GFP fluorescence was observed using a confocal laser scanning microscope (TCS SP8x; Leica Microsystems, Wetzlar, Germany).

Tissue-specific expression of OsMGR2 with immunostaining analysis

To investigate the tissue-specific expression of OsMGR2, transgenic rice lines carrying the *OsMGR2* promoter fused with *GFP* were generated. The 3138 bp of promoter region of *OsMGR2* (*proOsMGR2*) was amplified from rice genomic DNA using primers listed in *SI Appendix*, Table S3. Then the promoter fragment was

fused to the *GFP* fragment. The fused DNA was inserted into the pPZP2H-lac vector (7). The resulting construct was transformed into calluses (cv. Nipponbare) by *Agrobacterium*-mediated transformation (3). For observation, 21-d-old seedlings (T2) of the transgenic lines harboring *proOsMGR2-GFP* and non-transgenic rice (as a negative control) were used. Roots and shoot basal regions were sampled for immunostaining using an antibody against GFP. Node I and caryopses were sampled from soil-grown rice at the grain filling stage. The method for immunostaining was the same as described in Yamaji and Ma (8). The signal of fluorescence was observed with a TCS SP8x confocal laser scanning microscope (TCS SP8x; Leica Microsystems, Wetzlar, Germany).

Transport assay

The transport activity of OsMGR2 was examined using proteoliposomes. Recombinant baculovirus containing the full length of *OsMGR2* was cloned into Bac-to-Bac baculovirus expression systems (Invitrogen) according to the manufacturer's protocol. Expression and purification procedures followed the protocol as described previously (9). For the transport assay, an aliquot (containing 30 μg) of purified OsMGR2 was mixed with liposomes (500 μg) and frozen at −80°C for at least 10 min. The mixture was diluted with reconstitution buffer containing 20 mM MES-KOH (pH 6.0) and 0.1 M potassium acetate. Reconstituted proteoliposomes were pelleted by centrifugation at 200,000 *g* for 1 h at 4°C and then suspended in reconstitution buffer. Liposomes (10 mg/mL) containing 40% phosphatidylcholine, 30% phosphatidylethanolamine, 10% phosphatidylserine, and 20% cholesterol (by weight ratio) were prepared in buffer

containing 20 mM MES-KOH (pH 6.0) and 1 mM DTT as described previously (9). For Mg transport, reaction mixtures (130 µL) containing 0.75 µg of protein incorporated into proteoliposomes, 20 mM MES-KOH (pH 6.0) or 20 mM MOPS-KOH (pH 7.5), 0.1 M potassium acetate, 10 mM KCl and 1 mM $MgCl_2$ were incubated at 27°C. After 2 min of incubation, the transport assay was terminated by separating the proteoliposomes from the external mixture using centrifuge columns packed with Sephadex G-50 (fine). The Mg incorporated into the liposomes was determined by inductively coupled plasma-mass spectrometer (ICP-MS) as described below.

Phenotypic analysis of *osmgr2* mutants in hydroponic solution and soil. The WT rice and two independent *OsMGR2* knockout lines (T2, *osmgr2-1*, *osmgr2-2*) generated by CRISPR/Cas9 were used for phenotypic analysis. For hydroponic culture, de-husked seeds were sown in half-strength Kimura B supplemented with 2.5 mM Mg supply for 2 days. After germination, the plants were pre-cultured at 2.5 mM Mg for 16 days. Plants were then cultured in solutions containing 0.025, 0.25, or 2.5 mM Mg. In the treatment solution, 0.25 mM $MgSO_4$ was replaced with 0.25 mM Na_2SO_4, and $MgCl_2$ was supplied to generate different Mg concentrations. The treatment solution was renewed every 2 days. After 17 days, the plants were photographed. Roots were washed three times with 5 mM of $CaCl_2$ and separated from the shoots. Dry weight of the roots and shoots were recorded. Concentrations of mineral elements in the roots and shoots were determined as described below.

To measure the SPAD value (chlorophyl meter reading), WT and two mutants

(22-d-old) were grown at different Mg concentrations (0.01, 0.25, and 5 mM) as described above. After 12 days, the newest fully expanded leaf was subjected to SPAD value measurement using a SPAD-502 chlorophyll meter (Konika Minolta, Tokyo, Japan). Leaves were also photographed.

For soil culture, 30-d-old seedlings of both WT and knockout lines grown hydroponically were transplanted to pots containing 3.5 kg of soil, with three replicates. The soil, collected from a field at the Institute of Plant Science and Resources, Okayama University, was gray lowland soil with a pH of 6.5. Fertilizer (12/12/12 for N/P_2O_5/K) was applied at 3 g per pot with 50 g silica gel (Water Silica). Tap water was supplied daily, and a 2-cm water layer was maintained on the topsoil. Soil solution was collected nondestructively from the pots using a looped hollow fibre (Nakamura Corp., Kyoto, Japan) according to the procedures described before (10). Elemental concentrations in the soil solution were: macronutrient (mM): 7.60 Mg, 0.20 P, 0.44 K, 19.0 Ca, and micronutrient (μM): 510 Mn, 5,560 Fe, 0.15 Cu, and 1.6 Zn. At maturity (113 days after transfer to soil culture), plants were harvested and separated into brown rice, husk, rachis, peduncle, nodes I and II, leaf blade and sheath of flag leaf and leaf II, and internode II. The concentrations of mineral elements were determined by ICP-MS as described below. The distribution ratio was calculated as the element content in each organ divided by the total element content in all organs, multiplied by 100% (distribution = (element content in each organ/ total element content in all organs) X 100%)).

Labelling experiment with stable isotope ^{25}Mg

For a short-term labelling experiment with ^{25}Mg at the vegetative stage, 27-d-old seedlings of the WT and two knockout lines (T2) pre-cultured with 2.5 mM Mg were exposed to Mg-free nutrient solution for 2 days. The labeling experiment was conducted by exposing the plants to nutrient solution containing 25 μM ^{25}Mg, in the presence of 1 μM rubidium (Rb) and strontium (Sr). Rb and Sr were used as symplastic tracers and apoplastic tracers, respectively (11). After 2 d of exposure, plants were separated into root, shoot basal region, leaf 2-4, and other individual leaves. Accumulation of ^{25}Mg and other mineral elements were determined as described below.

For the stem-fed experiment, the WT and knockout lines (T2) were cultivated in soil pots until the grain-filling stage. The stem was cut below node II (internode III) and the cut end was exposed to nutrient solution containing 50 μM ^{25}Mg in the presence of 1 μM Rb and Sr. After 1 day, spikelet, rachis, peduncle, node I, flag leaf, internode I, node II, and leaf II were harvested and subjected to determination of ^{25}Mg with stable isotope mode and other elements by ICP-MS (7700X; Agilent Technologies, Santa Clara, CA, USA) as described below. The experiment was conducted in a growth chamber at 30°C.

Characterization of grain size and weight

To compare the size and weight of grains between the WT and mutants, we used the SmartGrain software (12). Grain size was measured for 200 grains per plant of WT and mutants, with three replicates. Thousand-grain weight was measured using a gauge.

Evaluation of eating quality

To evaluate eating quality, two instrumental methods significantly correlated with sensory test score were used (12). Eating quality scores were measured using a Cooked Rice Taste Analyser STA1A (Satake Co. Ltd.) according to the previous study, based on the amount of near-infrared light reflected and transmitted by cooked rice grains. Six measurements were made for one replicate. Physical properties of cooked grains, including hardness and stickiness of grain surface and whole grains were measured using a Tensipresser MyBoy texture analyzer (Takemoto Electric Co.) with the high-compression/low-compression method and the continuous progressive compression method (13). Twenty grains per one replicate were measured.

Element determination in plant samples

All harvested samples were dried in an oven at 70°C for at least 2 days. Dried samples were transferred to glass tubes with concentrated HNO_3 (61%) and heated on a metal bath at temperature up to 140°C until the solution became clear. The concentration of mineral elements in the digested solution was determined by ICP-MS (7700X; Agilent Technologies, Santa Clara, CA, USA). Yttrium (Y) and Tellurium (Te) were used as internal standards for all samples. Certified rice standard materials were used to ensure the accuracy. Calibration was performed using multi-element standard solutions covering the expected concentration ranges. For the determination of ^{24}Mg and ^{25}Mg, isotope mode was employed. ^{24}Mg and ^{25}Mg were measured separately and distinguished based on their mass-to-charge ratio (m/z).

RNA-seq analysis

Immature grains were sampled from both the WT and *osmgr2-1* grown in soil pots. At the grain filling stage (8 d after filling), the grain coat was carefully peeled by tweezers, and the immature grain was subjected to RNA extraction as described above. RNA-seq was performed using a DNBSEQ-G400FAST (MGI, Kobe, Japan) for paired-end sequencing. An average of 30-40 million stranded paired-end (2 × 150 bp) sequences was obtained per sample. Four to six grains from each line were combined as one sample, with three biological replicates per line. Sequences were mapped to the IRGSP-1.0 rice reference genome (https://rapdb.dna.affrc.go.jp) using STAR-Salmon with the nf-core/rnaseq pipeline (https://nf-co.re/rnaseq/) with default parameters. Genes with total TPM (Transcripts Per Million) values > 40 across all biological replicates were used for differential expression analysis. Differentially expressed genes (DEGs) were identified using the edgeR (http://www.bioconductor.org/packages/release/bioc/html/edgeR.html) software package in R, with a false discovery rate (FDR) < 0.05 and |fold change| ≥ 2.0. Gene ontology (GO) and Kyoto Encyclopedia of Genes and Genomes (KEGG) pathway analyses were performed using the OmicShare tools, a free online platform for data analysis.

Statistical analysis of data

Statistical analyses were performed using Student's *t*-test or one-way ANOVA followed by Tukey's test. The significance levels were defined as: *, $P < 0.05$; **, $P < 0.01$.

Data Availability

The RNA-seq data generated in this study have been deposited in the DDBJ BioProject under accession number PRJDB40261. All data supporting the findings of this study are available in the article, SI Appendix, information and dataset files.

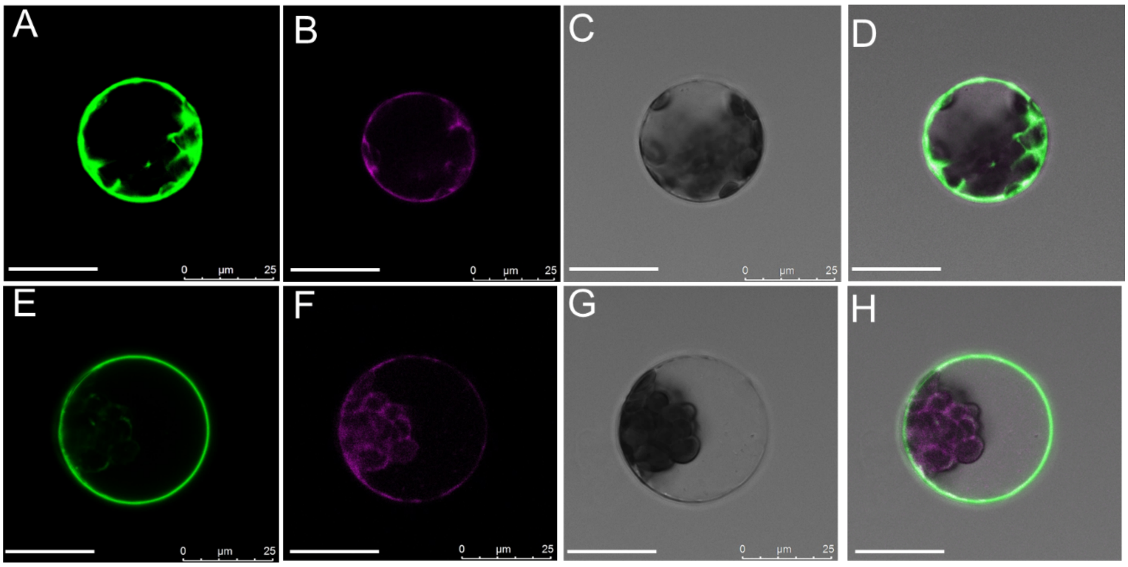

Fig. S1 Subcellular localization of OsMGR2 in rice protoplasts. (*A*-*H*) Subcellular localization of OsMGR2 in rice protoplasts. Plasmids of *GFP* alone (*A*-*D*) or *OsMGR2* fusion (*E*-*H*) were transiently expressed in rice protoplasts together with DsRed as a cytosolic and nuclear marker. Fluorescence signals from GFP (*A*, *E*), DsRed (*B*, *F*), bright field (*C*, *G*), and the merged images (*D*, *H*) are shown. Scale bar = 25 µm (*A*-*H*).

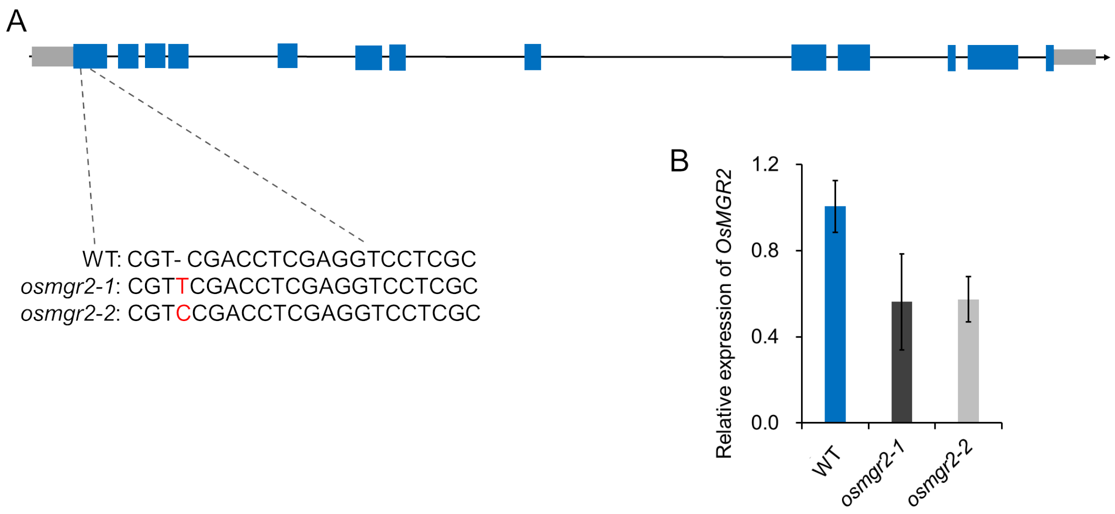

Fig. S2 Target sites used for generation of *OsMGR2* knockout lines with CRISPR/Cas9. (*A*) Schematic diagram of the *OsMGR2* gene structure. Gray boxes represent UTR regions, blue boxes represent exons, and the lines between boxes represent introns. (*B*) Relative expression of *OsMGR2* in knockout lines.

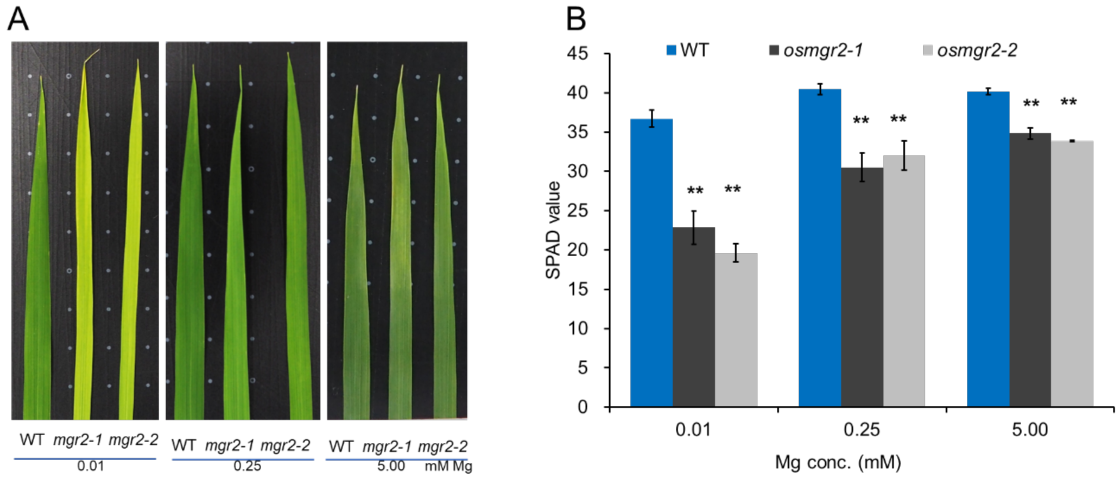

Fig. S3 Effect of *OsMGR2* knockout on mineral element accumulation in roots at the vegetative stage. (*A*-*G*) Concentration of P (*A*), K (*B*), Ca (*C*), Mn (*D*), Fe (*E*), Cu (*F*), and Zn (*G*) in roots. 16-d-old plants pre-cultured at 2.5 mM Mg for 16 days were exposed to nutrient solutions containing 0.025, 0.25, or 2.5 mM for 17 days. Data represent means ± SD of four biological replicates (n = 4). * or ** indicates significant difference compared with WT ($P < 0.05$ or $P < 0.01$, one-way ANOVA followed by Tukey's test).

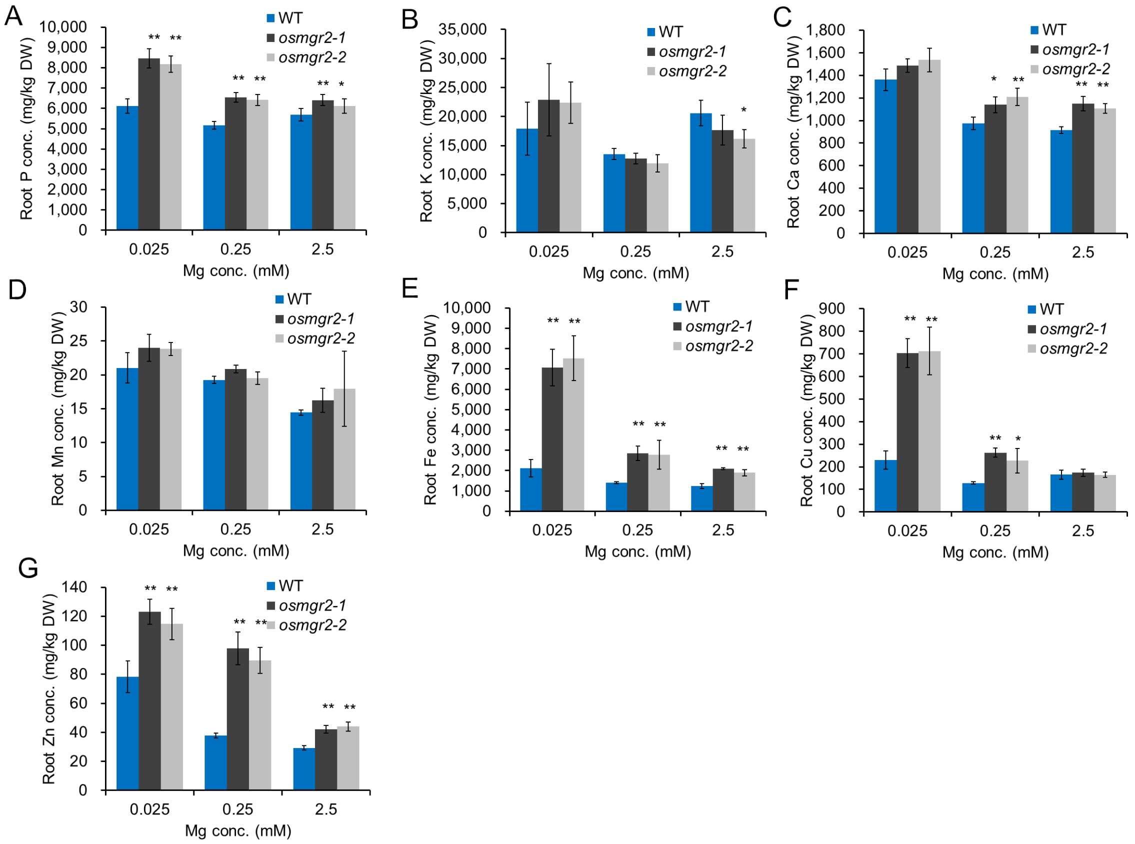

Fig. S4 Effect of *OsMGR2* knockout on mineral element accumulation in shoots at the vegetative stage. (*A*-*G*) Concentration of P (*A*), K (*B*), Ca (*C*), Mn (*D*), Fe (*E*), Cu (*F*), and Zn (*G*) in shoots. 16-d-old plants pre-cultured at 2.5 mM Mg for 16 days were exposed to nutrient solutions containing 0.025, 0.25, or 2.5 mM for 17 days. Data represent means ± SD of four biological replicates. * or ** indicates significant difference compared with WT ($P < 0.05$ or $P < 0.01$, one-way ANOVA followed by Tukey's test).

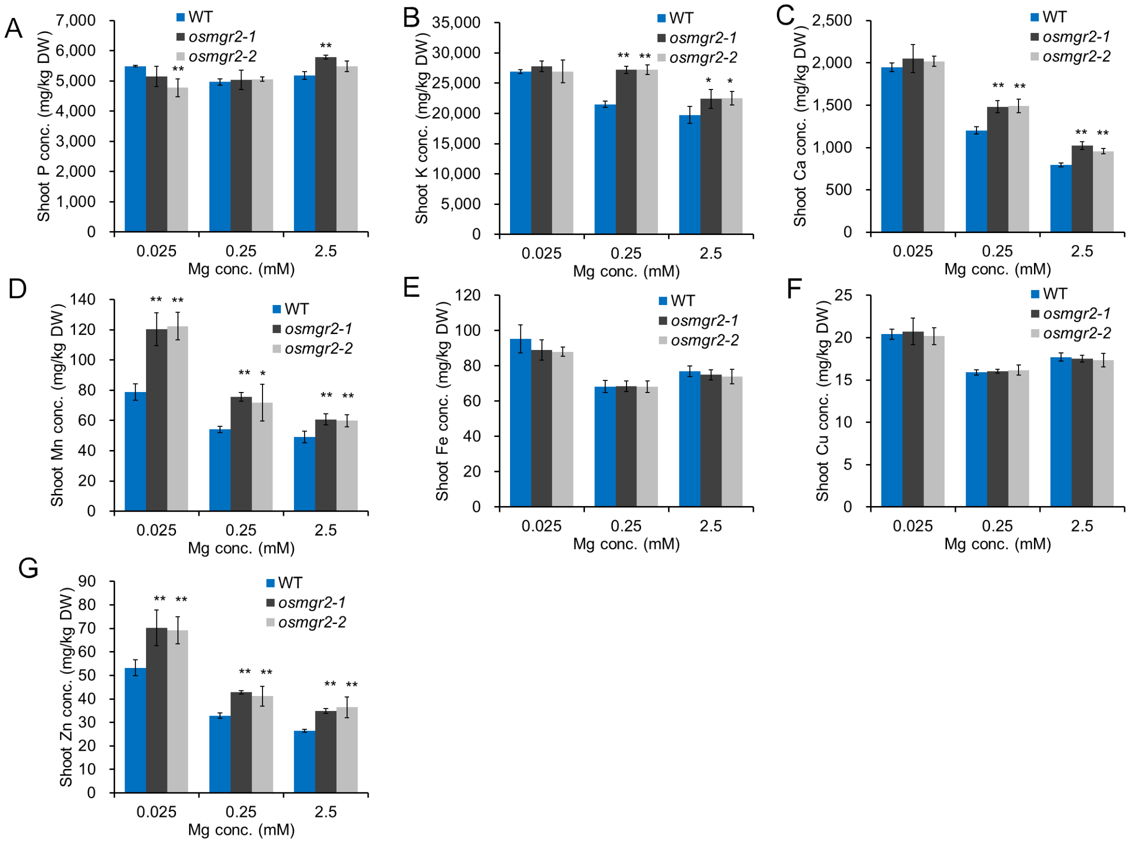

Fig. S5 Comparison of SPAD value between *OsMGR2* knockout lines and WT. (*A*) Chlorosis in the newest fully expanded leaf. (*B*) SPAD values of the newest fully expanded leaf. 22-d-old seedlings of the WT and two knockout lines pre-cultured at 2.5 mM Mg were treated with different Mg concentrations (0.01, 0.25, and 5 mM) for 12 days. Data represent means ± SD of four biological replicates (n = 4). ** indicates significant difference compared with WT ($P < 0.01$, one-way ANOVA followed by Tukey's test).

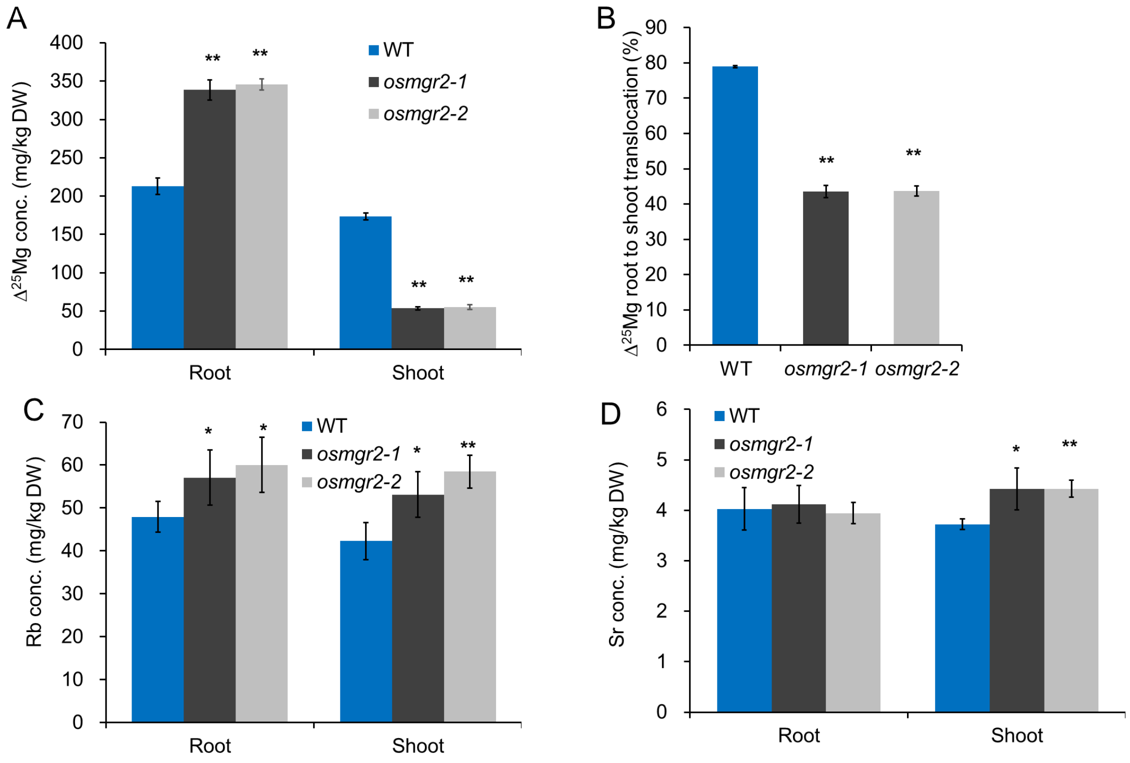

Fig. S6 Short-term labeling experiment with ^{25}Mg at the vegetative stage. (*A*) Concentration of Δ^{25}Mg in roots and shoots. (*B*) Root-to-shoot translocation of Δ^{25}Mg. (*C*, *D*) Concentrations of Rb (*C*) and Sr (*D*). 27-d-old seedlings of the WT and two knockout lines pre-cultured at 2.5 mM Mg were exposed to Mg-free nutrient solution for 2 days, followed by exposure to nutrient solution containing 25 μM ^{25}Mg in the presence of 1 μM rubidium (Rb) and strontium (Sr). After 2 days, the roots and shoots were harvested separately and analyzed for mineral element concentrations by ICP-MS with isotope mode. Data represent means ± SD of three biological replicates (n = 3). * or ** indicates significant difference compared with WT ($P < 0.05$ or $P < 0.01$, one-way ANOVA followed by Tukey's test).

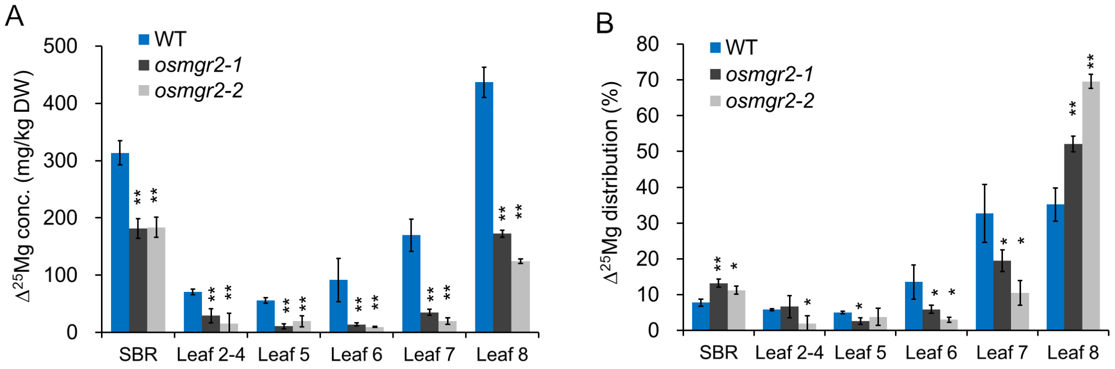

Fig. S7 Organ-dependent distribution of ^{25}Mg at the vegetative stage. (*A*, *B*) Concentration (*A*) and distribution (*B*) of ^{25}Mg in different organs. 27-d-old seedlings of the WT and two knockout lines pre-cultured at 2.5 mM Mg were exposed to Mg-free nutrient solution for 2 days, followed by exposure to nutrient solution containing 25 μM ^{25}Mg. After 2 days, roots and shoots were harvested separately and analyzed for ^{25}Mg concentration by ICP-MS with isotope mode. SBR, shoot basal region. Data represent means ± SD of three biological replicates (n = 3). * or ** indicates significant difference compared with WT ($P <$ 0.05 or $P < 0.01$, one-way ANOVA followed by Tukey's test).

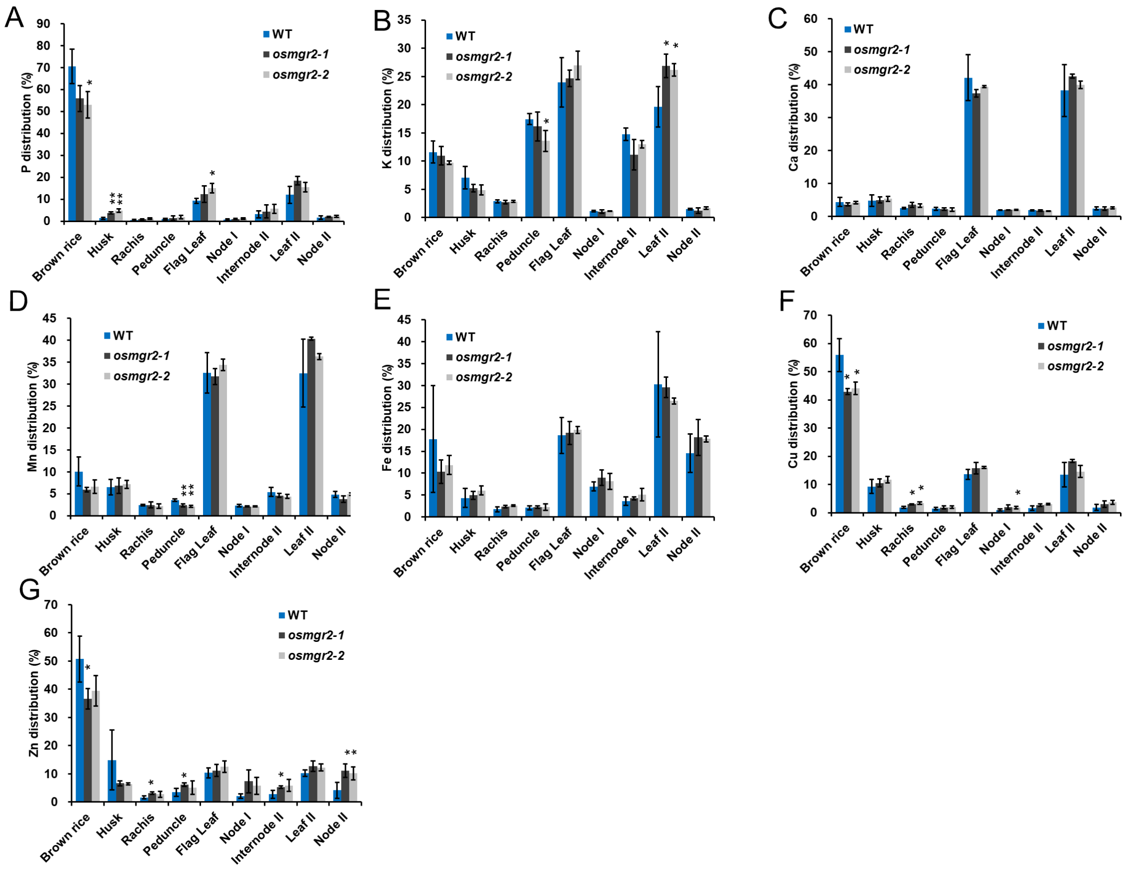

Fig. S8 Effect of *OsMGR2* knockout on mineral element distribution at the reproductive stage. (*A*-*G*) Distribution of P (*A*), K (*B*), Ca (*C*), Mn (*D*), Fe (*E*), Cu (*F*), and Zn (*G*) in different organs. Wild-type (WT) rice and knockout lines (*osmgr2-1*, *osmgr2-2*) were grown in pot soil under flooded conditions until maturity. At harvest, different organs were collected separately and analyzed for mineral element concentration by ICP-MS. Data represent means ± SD of three biological replicates (n = 3). * or ** indicates significant difference compared with WT ($P < 0.05$ or $P < 0.01$, one-way ANOVA followed by Tukey's test).

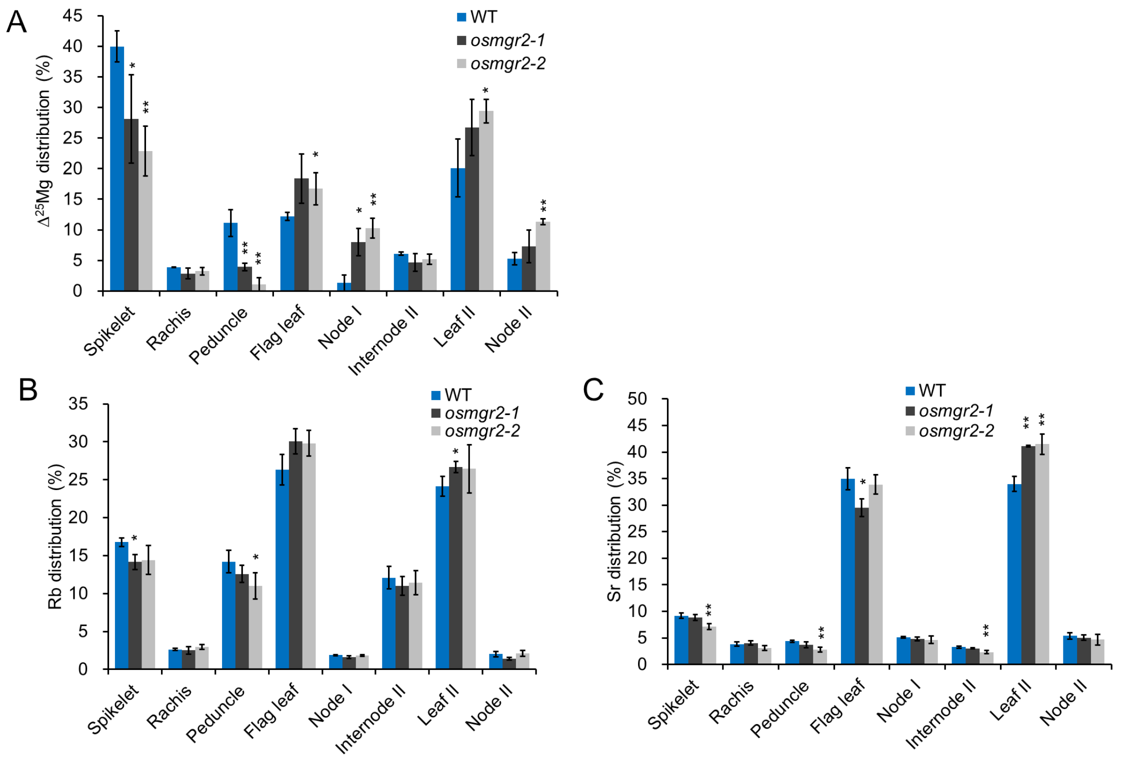

Fig. S9 Short-term stem-feeding experiment with stable isotope ^{25}Mg at the grain filling stage

(*A*-*C*) Distribution of Δ^{25}Mg (*A*), Rb (*B*), and Sr (*C*) in different organs of rice. Wild-type (WT) rice and two knockout lines (*osmgr2-1*, *osmgr2-2*) were cultivated in soil pots. At the grain-filling stage, stems were cut below node II, and the cut ends were exposed to nutrient solution containing 50 μM ^{25}Mg in the presence of 1 μM Rb and Sr. After 24 h, different organs were harvested and analyzed for mineral element concentration by ICP-MS. Data represent means ± SD of four biological replicates (n = 4). * or ** indicates significant difference compared with WT ($P < 0.05$ or $P < 0.01$, one-way ANOVA followed by Tukey's test).

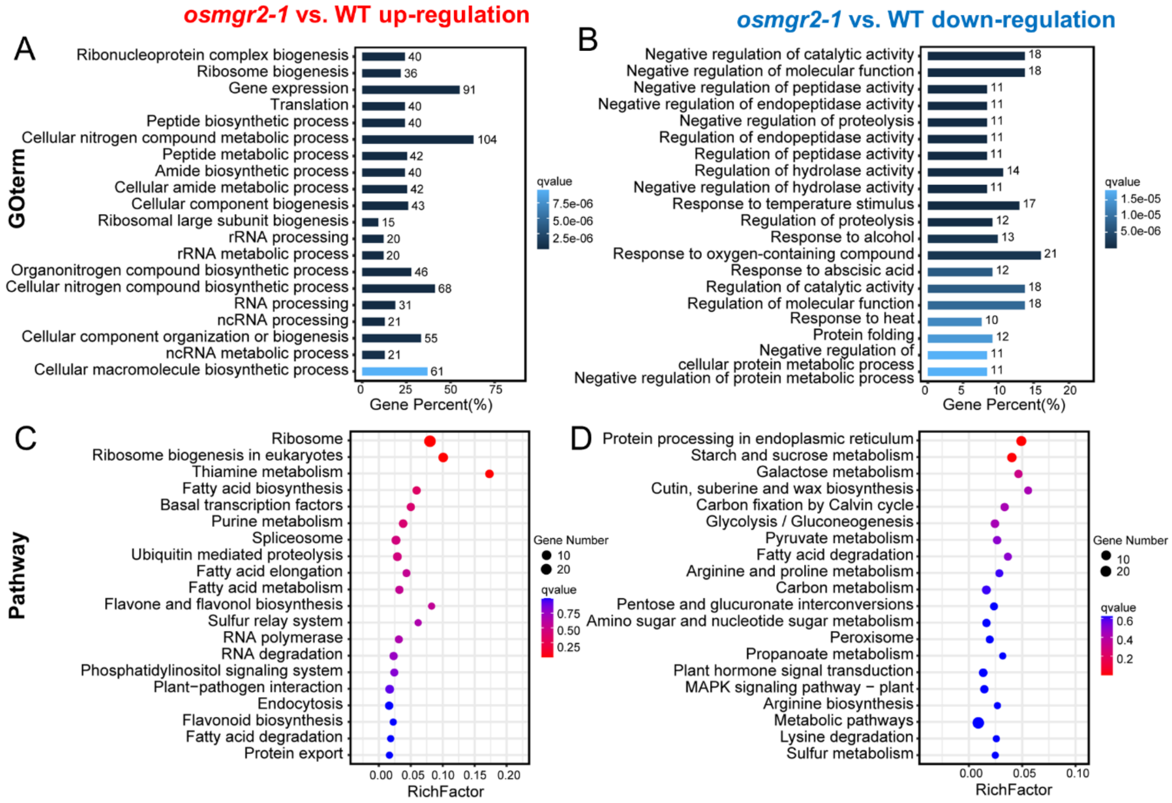

Fig. S10 Transcriptome analysis of immature grains. (*A*, *B*) Gene ontology (GO) analysis of genes up-regulated (*A*) and down-regulated (*B*) in *osmgr2-1* mutant. (*C*, *D*) KEGG pathway enrichment analysis of up-regulated (*C*) and down-regulated (*D*) genes. Immature grains were sampled from wild-type (WT) rice and *osmgr2-1* grown in soil pots at the grain filling stage (8-d after flowering). After carefully removing the grain coat with forceps, immature grain was collected for RNA-seq analysis. (*A*-*D*) Bar plots (*A*, *B*) and bubble chart (*C*, *D*) showing the top 20 enriched GO term and KEGG enrichment pathways, respectively.

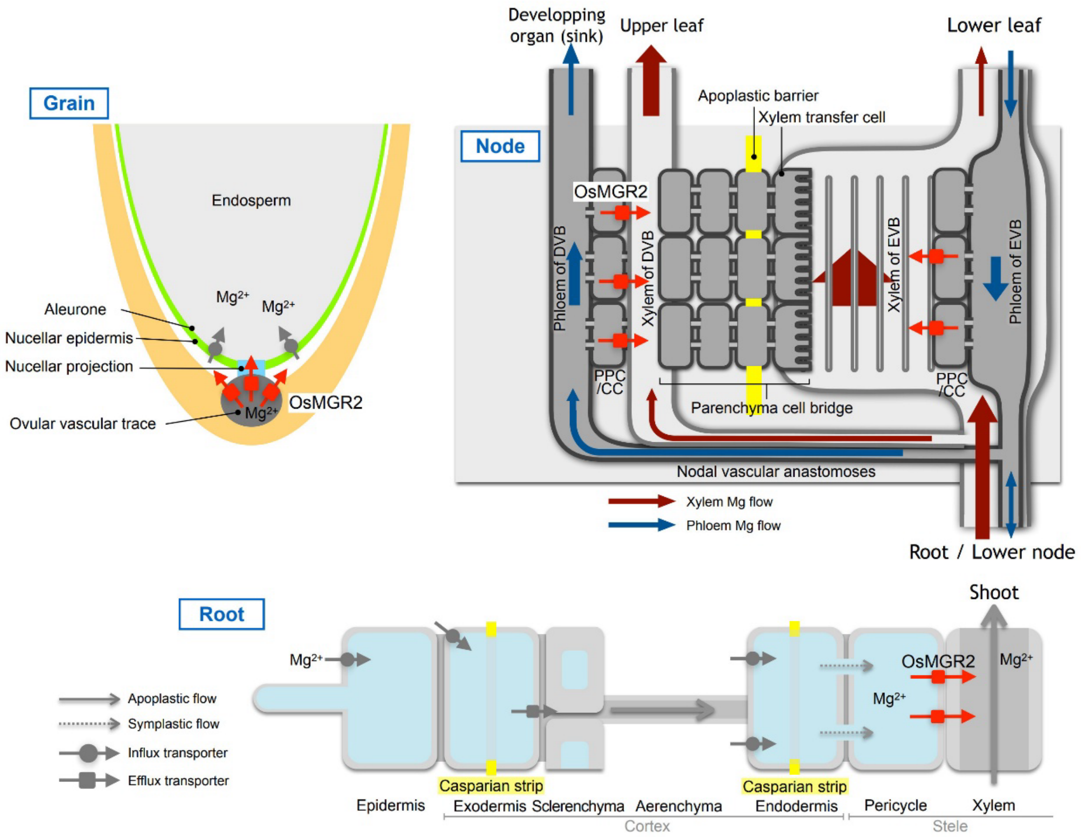

Fig. S11. Schematic representation of the roles of OsMGR2 in magnesium (Mg) accumulation in different rice organs. OsMGR2 plays multiple roles in Mg transport in rice. First, OsMGR2 expressed in the root stele region mediates root-to-shoot translocation of Mg. Second, OsMGR2 expressed in the phloem regions of both enlarged vascular bundles and diffuse vascular bundles of nodes is required for preferentially Mg delivery to the second newest organs, Third, OsMGR2 expressed at the ovular vascular (OVT) mediates Mg export from maternal vascular tissues of the caryopsis to the grain, a process crucial for grain development and eating quality in rice.

Gene ID	Gene name	FPKM				
		EVB xylem without BS	EVB xylem with BS	EVB+DVB phloem	DVB	PCB
Os03g0125800	***OsMGR2***	0.0	0.0	5.8	19.3	1.9
Os03g0751100	*OsOPT7*	132.5	140.1	4.9	3.6	13.0

Table S1 Tissue-specific expression of *OsMGR2* in node I of rice. Different tissues of node I were isolated by laser microdissection (LMD), followed by RNA-seq analysis. Enlarged vascular bundle (EVB) tissues outside the bundle sheath (BS) (with BS) and inside the BS (without BS) were further dissected. FPKM values for each gene in different tissues are shown. Abbreviation: DVB, diffuse vascular bundle; PCB, parenchyma cell bridge.

Gene ID	Gene name	FPKM		
		Rachilla	Ovuller Vascular	Scutellum
Os03g0125800	***OsMGR2***	13.0	43.6	8.6
Os03g0751100	*OsVIT2*	116.0	11.3	31.3

Table S2 Tissue-specific expression of *OsMGR2* in immature grain. Different tissues including rachilla, ovular vascular and scutellum from immature grain were isolated from immature grains by laser microdissection (LMD), followed by RNA-seq analysis. FPKM values for each gene in different tissues are shown.

Primer name	Forward (5'-3')	Reverse (5'-3')	Purpose
OsMGR2-criA	GTTGCGAGGACCTCGAGGTCGACG	AAACCGTCGACCTCGAGGTCCTCG	CRISPR/cas9 system
OsMGR2-criB	GTTGGCTGGTTCTTGACGACCGGC	AAACGCCGGTCGTCAAGAACCAGC	
OsMGR2-A/B-seq	GTCGTACTGAGCATCTGAGCTC	AATATCGGCAGCGCCTG	Sequencing of mutants
RRT-OsMGR2	CCACTTCCAAGCTATTCATTGG	TGATAATGATTTGCCCAGAGGT	qRT-PCR
RRT-Histone H3	GGTCAACTTGTTGATTCCCCTCT	AACCGCAAAATCCAAAGAACG	
GFP-OsMGR2	GGTCCGGAATGTCGCAGCATGCGG	AAGGAAAAAAGCGGCCGCTTACGAATTCCTT GAGACCTGA	Subcellular localization
OsMGR2-GFP	GGTCCGGAATGTCGCAGCATGCGG	GGTCCGGACGAATTCCTTGAGACCTGAGT	
promoter-OsMGR2	CGGGGTACCTGCAAATGGAAAATGAC ATGCCCCA	CGCGGATCCCTCCCCCCCGCCGCC	Promoter amplification
pMGR2-920F	GCTGGCTACAAATCTGTAAGAGC	None	Sequencing
pMGR2-1960F	AGACCGATATAATTAACCATGCAT	None	
pMGR2-2500F	AGTCACCACCACCGGCA	None	
pMGR2-2800F	ACAGCCCCTCTGGGGAG	None	
pMGR2-1110R	None	GATAAAGGAAGAGAGAAGAACAGCT	
pMGR2-2080R	None	TCAGTCAAAAGGAAAGTTACTTTGA	
OsMGR2-800F	GGATGAAGTGCCCATTCGA	None	
OsMGR2-900R	None	ACCACCACAGCCATGTGACT	

Table S3 Primer sequences used in this study.

R eferences

1. J. F. Ma, K. Tamai, M. Ichii, G. F. Wu, A rice mutant defective in Si uptake. *Plant Physiol.* **130,** 2111–2117 (2002).
2. M. Mikami, S. Toki, M. Endo, Comparison of CRISPR/Cas9 expression constructs for efficient targeted mutagenesis in rice. *Plant Mol. Biol.* **88,** 561–572 (2015).
3. Y. Hiei, S. Ohta, T. Komari, T. Kumashiro, Efficient transformation of rice (*Oryza sativa* L.) mediated by Agrobacterium and sequence analysis of the boundaries of the T-DNA. *Plant J.* **6,** 271–282 (1994).
4. A. Sasaki, N. Yamaji, N. Mitani-Ueno, M. Kashino, J. F. Ma, A node-localized transporter OsZIP3 is responsible for the preferential distribution of Zn to developing tissues in rice. *Plant J.* **84,** 374–384 (2015).
5. A. Sasaki, N. Yamaji, K. Yokosho, J. F. Ma, Nramp5 is a major transporter responsible for manganese and cadmium uptake in rice. *Plant Cell* **24,** 2155–2167 (2012).
6. S. Chen *et al.*, A highly efficient transient protoplast system for analyzing defence gene expression and protein–protein interactions in rice. *Mol. Plant Pathol.* **7,** 417–427 (2006).
7. T. Fuse, T. Sasaki, M. Yano, Ti-plasmid vectors useful for functional analysis of rice genes. *Plant Biotechnol.* **18,** 219–222 (2001).
8. N. Yamaji, J.F. Ma, Spatial distribution and temporal variation of the rice silicon transporter Lsi1. *Plant Physiol.* **143,** 1306–1313 (2007).
9. T. Miyaji *et al.*, AtPHT4; 4 is a chloroplast-localized ascorbate transporter in Arabidopsis. *Nature Commun.* **6,** 5928 (2015).

10. D. Ueno, F-J. Zhao, R. Shen, J. F. Ma, Cadmium and zinc accumulation by the hyperaccumulator *Thlaspi caerulescens* from soils enriched with insoluble metal compounds. *Soil Sci. Plant Nutri.* **50**, 511–515 (2004).
11. H. Kuppelwieser, U. Feller, Transport of Rb and Sr to the ear in mature, excised shoots of wheat: effects of temperature and stem length on Rb removal from the xylem. *Plant Soil* **132,** 28–288 (1991).
12. T. Tanabata, T. Shibaya, K. Hori, K. Ebana, M. Yano, SmartGrain: high-throughput phenotyping software for measuring seed shape through image analysis. *Plant Physiol.* **160,** 1871–1880 (2012).
13. K. Hori, K. Suzuki, K. Iijima, K. Ebana, Variation in cooking and eating quality traits in Japanese rice germplasm accessions. *Breed. Sci.* **66,** 309–318 (2016).
